# Predictors of cognitive change in cognitively healthy older women in Panama: the PARI-HD study

**DOI:** 10.3389/fgwh.2024.1353657

**Published:** 2024-06-13

**Authors:** Diana C. Oviedo, Adam E. Tratner, Sofía Rodríguez-Araña, Alcibiades E. Villarreal, Giselle Rangel, María B. Carreira, Gabrielle B. Britton

**Affiliations:** ^1^ Centro de Neurociencias y Unidad de Investigación Clínica, Institiuto de Investigaciones Científicas y Servicios de Alta Tecnología (INDICASAT-AIP), Panama City, Panamá; ^2^ Escuela de Psicología, Universidad Santa María la Antigua (USMA), Panama City, Panamá; ^3^ Sistema Nacional de Investigación (SNI) SENACYT, Panama City, Panamá; ^4^ Florida State University, Panama City, Panamá

**Keywords:** Hispanic, cognition, cardiovascular, depressive symptoms, health behaviors, older adults, Latin America, longitudinal studies

## Abstract

**Background:**

Evidence suggests that a combination of biological and social factors influence risk of dementia differently for women and men. In healthy older women, several factors may contribute to changes in cognition.

**Objective:**

Describe the characteristics associated with variation in cognition in a sample of cognitively healthy older Panamanian women.

**Methods:**

The study includes cross-sectional analyses of cognitive domains at baseline (*n *= 357) and 17-month (SD = 2.0) follow-up (*n *= 200) for women aged 60 years and older enrolled in the Panama Aging Research Initiative-Health Disparities (PARI-HD) study. Instruments included clinical questionnaires, physiological measures, and a neuropsychological test battery assessing global cognition and seven cognitive domains. Multiple regression analyses examined the associations between demographic and clinical characteristics and cognition at baseline. Repeated measures analyses were used to investigate changes in cognition from baseline to follow-up.

**Results:**

On average, participants were 68.6 years of age (SD = 5.9) with 16.1 years of education (SD = 4.7). Age, income, and education showed robust associations with baseline cognition. Subjective cognitive impairment was associated with lower performance in global cognition, verbal learning, and memory domains. Only performance in the attention domain decreased at follow-up, and subjective health state and depressive symptoms significantly predicted the change in attention.

**Discussion:**

Our study findings contribute to the investigation of cognitive health in older Hispanic women and to the understanding of sociodemographic and health-related factors associated with cognitive decline and the progression to cognitive impairment and dementia.

## 1 Introduction

Population aging is occurring in all countries of the world, but with a more rapid rate of increase in low- and middle-income countries (LMIC) (1). Countries in the Latin American and Caribbean (LAC) region are experiencing one of the fastest rates of population aging (2, 3). Estimates show that by 2050, the number of adults over 60 years of age in the LAC region will double (1). An increase in the number of aging individuals is associated with a rise in mild cognitive impairment (MCI) and dementias such as Alzheimer's disease (AD). The prevalence of dementia in LAC has been estimated at 10% (4) analogous to more developed regions but with a more accelerated growth. Projections indicate that by 2050, the number of people living with dementia will increase by 449% (reaching 978,000 cases). In Central America, Panama is projected to have the 3rd highest percentage increase in dementia cases (273%) due to socioeconomic and health factors (5). Although Panama is classified as a middle-high income country, it is globally ranked as one of the most unequal nations in terms of wealth distribution and is the third most unequal country in the LAC region (6, 7). A large proportion of the population cannot access high quality public services, such as health care, education, and sanitation. Furthermore, Panama's public education and healthcare systems are constrained by limited funding, precarious infrastructure, and frequent shortages of workers. These factors are implicated in the high prevalence of chronic illnesses, such as hypertension, diabetes, and obesity, as well as health disparities across the population, given that many people do not receive timely and effective healthcare services or adequate health education (6, 8, 9).

Aging is a normal process involving physiological and psychological changes. Research has shown that although most cognitive functions decline with age, these can decrease at different rates (10). Cognitive domains such as episodic memory, processing speed, executive function, and verbal fluency tend to decline more rapidly and at an earlier age (11–13), whereas other aspects of cognition such as vocabulary and general knowledge, remain more stable and can even improve over time (14, 15). Moreover, there is interindividual variability regarding cognitive trajectories and decline. Some individuals exhibit high cognitive performance, comparable to younger adults, while others experience a steeper and more rapid decline, that could progress to cognitive impairment and dementia (14). Changes in cognition also vary between women and men. In dementia-free women, some studies reveal that cognition levels remain stable for longer periods of time relative to men (16–18), in line with cross-sectional studies that suggest women perform better in some cognitive tasks than men (18–20). Nonetheless, the overall estimated prevalence for dementia is higher for women than men (5), although sex differences in the incidence of dementia are less clear, and may vary across geographical regions (21, 22). These contradictory findings call for further research in women's cognitive health.

Evidence suggests that a combination of biological and social factors influence risk of dementia differently for women and men (23, 24). In healthy older women, several factors may contribute to changes in cognition. Longitudinal studies show that sociodemographic characteristics (e.g., age, and sex), socioeconomic status (e.g., education, income, employment), physical and mental health-related determinants (e.g., chronic illnesses, BMI, depression) and daily habits (e.g., sleeping, physical activity, and smoking) contribute to maintenance or decline of cognitive function (25, 26). Depression and stress in women are also notable risk factors associated with cognitive decline (27, 28). Several studies show that depression is associated with poorer cognitive functioning and faster cognitive decline over time, even for individuals with low to moderate symptoms (29–33). In addition, some research suggests that cardiovascular risk factors (e.g., high systolic blood pressure, obesity) decrease cognitive function (34–37). However, most research has been carried out in predominantly Caucasian populations, which limits the generalizability of the results to other populations (38). In LAC countries, where the prevalence of chronic vascular diseases is increasing (39) these risk factors are of special relevance.

Research on aging women is particularly limited in LAC countries. Some studies that include both men and women tend to overlook gender differences in their analyses, therefore limiting findings on women's cognitive health (4, 40). In Panama, women face unique economic, educational and health obstacles (41). Women perform twice as much domestic labor, earn lower wages, and are less likely to be employed full-time compared to men. This may stem, in part, from the fact that more than 80% of caregivers in Panama are women, which constrains women’s career prospects. Consequently, women are more likely to pursue informal and part-time employment, characterized by lower wages and job insecurity (42). Furthermore, many women experience barriers to accessing and completing education due to poverty, early pregnancies, and gender-based violence (43, 44). The Panama Aging Research Initiative- Health Disparities (PARI-HD) program has studied cohorts of older adults for over a decade, and established the first longitudinal aging study in the country as well as one of the few in the broader LAC region. In the present study, the main objective was to examine the characteristics associated with variation in cognition using a sample of cognitively healthy older Panamanian women assessed at two time points.

## 2 Methods

### 2.1 Participants and procedure

The study included women aged 60 years and older enrolled in the PARI-HD study, an ongoing community-based longitudinal study of the factors affecting cognitive aging in older adults in Panama. Participants were recruited from the community using convenience sampling. The research team used advertisements on social media platforms that included a description of the study objectives and inclusion criteria. The study was also divulged in public outreach events. Interested adults who met inclusion criteria were recruited. At baseline, participants were literate, dementia-free and community-dwelling, as per inclusion criteria, and enrolled after providing informed consent. The study protocol was approved by the Institutional Bioethics Committee of the Caja del Seguro Social (P-083-16). Participants underwent clinical interviews, physical and cognitive assessments. Data included in this manuscript were collected between October 2016 and March 2020. The first follow-up visit began April 2018. All in-person visits were suspended due to the COVID-19 pandemic. Here we include cross-sectional data for female participants at baseline (*n *= 357) and those who had complete evaluations at first follow-up (*n *= 200). The time elapsed between visits was 17 months (SD = 2.0).

### 2.2 Measures

#### 2.2.1 Clinical interviews and instruments

Evaluations were conducted in Spanish by health professionals as well as medical, undergraduate, and graduate students trained by a multidisciplinary group of health specialists. A questionnaire based on the 15-item Subjective Memory Complaints Questionnaire (45) was used to assess subjective memory complaints. The European Quality of Life Health Questionnaire (EQ-5D-3l) (39, 46) was used to evaluate subjective health status. The measure includes a visual analogue scale where health is rated on a scale from 0 (worse imaginable health) to 100 (best imaginable health). Independence in instrumental (IADL) and basic (BADL) activities of daily living was measured with Lawton and Brody (47) and Katz Index (48), respectively. For both IADL and BADL higher scores indicate higher function and independence. The Spanish version of the 15-item Geriatric Depression Scale (GDS-15) (49) was used to screen for depressive symptoms.

Other measures included waist circumference, body mass index (BMI; kg/m^2^) and blood pressure. A cardiovascular risk (CVR) score (range 0–4) was calculated based on four common risk factors (50, 51): BMI with a cutoff score of less risk ≤30 kg/m or more risk >30 kg/m; blood pressure (systolic) with a cutoff of ≤140 mm Hg or >140 mm Hg; smoking (current or ever smoked) with a score of 0 (never smoked) or 1 (current/past smoker); and physical activity, which was measured through self-reported responses to the question: “Which of the following best describes your level of physical activity” and given the following options: (a) vigorous activity for at least 30 min 3 times a week; (b) moderate activity at least 3 times a week; and (c) rarely active, prefers sedentary activities (52). This item was scored positive if participants selected the last option.

#### 2.2.2 Neuropsychological testing

The neuropsychological test battery included measures of global cognition and seven cognitive domains: (1) attention, (2) executive function, (3) verbal learning, (4) memory, (5) language, (6) visuospatial abilities and (7) processing speed. Test scores were converted to *z* scores and averaged for each of the cognitive domains. Global cognition was measured by the 30-item Spanish version of the Mini-Mental State Examination (MMSE) (53) adjusted for age and education. The attention domain comprised the Trail Making Test Form A (TMT A) (54), and direct and inverse digit span (55). Executive function was evaluated with the TMT Form B (54), Phonetic Verbal Fluency Test (56), and INECO Frontal Screening (57). To assess verbal learning, the Consortium to Establish a Registry for Alzheimer's Disease Word List Memory Task (CERAD) (58) and the immediate recall for the Logical Memory subtest from the Wechsler Memory Scale (59) were used. The memory domain was measured with the recall forms of CERAD (58) and the Wechsler Memory Scale subtest on Logical Memory (59). Language was assessed with Semantic and Phonetic Verbal fluency tests (56) and Boston Vocabulary Test (60). Visuospatial abilities were measured with Clock Drawing Test (CDT) (61) in both forms, copy and draw to the order. Lastly, the time taken to complete the TMT A (seconds) was used as a measure of processing speed.

#### 2.2.3 Statistical analyses

Analyses were conducted using SPSS version 29. Descriptive analyses examined sample characteristics in relation to baseline and follow-up. Means and standard deviations were calculated for variables using a continuous scale of measurement, and frequencies and percentages were used to summarize categorical variables. Repeated measures analyses, McNemar's tests, or marginal homogeneity tests compared individuals at baseline and follow-up for each variable. A series of linear regressions explored the associations between demographic and clinical factors, and neuropsychological test scores for eight cognitive domains at baseline. Repeated measures analyses of covariance (ANCOVA) were used to investigate changes in cognition from baseline to follow-up. Eight simultaneous multiple regression analyses examined the associations between demographic and clinical characteristics and cognition at baseline. Cases with missing data were excluded from statistical analyses via listwise deletion.

## 3 Results

### 3.1 Descriptive analyses

Demographic and clinical characteristics are summarized in Table 1. At baseline, participants were 68.6 years of age (SD = 5.9) with 16.1 years of formal education (SD = 4.7). Most participants (60.1%) reported incomes in the upper quartile (*M* = 1,201–1,600 USD). Overall subjective health ratings were high (*M* = 86.3/100, SD = 12.6), although many participants reported subjective cognitive impairment (43.4%). BMI measurements (*M *= 27.3 kg/m^2^, SD = 4.9) indicated that many participants were overweight (40%) or obese (27%), and the most reported chronic illness was hypertension (51.5%). According to the Fried criteria (45) most participants were pre-frail (59.3%) and 9% were frail. The total number of chronic illnesses, medications, and the prevalence of subjective cognitive decline increased at follow-up. Moreover, performance declined in global cognition, learning, long-term memory, attention, and executive function from baseline to follow-up (see Table 2).

**Table 1:** Demographic and clinical characteristics of study participants.

Variable	Visit 1 (*n *= 357)	Visit 2 (*n *= 200)	*P* value
	*n* (%)/*M* (SD)	*n* (%)/*M* (SD)	
Age (years)	68.6 (5.9)	70.0 (5.9)	*p* < .001
Age group			*p* < .001
60–69	221 (61.9%)	116 (58.0%)	
70–79	111 (31.1%)	72 (36.0%)	
>80	25 (7%)	12 (6.0%)	
Marital status			*p* = .375
Partnered	197 (55.2%)	121 (60.8%)	
Not partnered	160 (44.8%)	78 (39.2%)	
Education (years)	16.1 (4.7)	16.2 (4.7)	*p* = .463
Highest education attained			*p* = .048
Incomplete primary school	4 (1.1%)	1 (0.5%)	
Completed primary school	44 (12.3%)	43 (21.5%)	
Completed high school	96 (26.9%)	33 (16.5%)	
University or higher	213 (59.7%)	123 (61.5%)	
Monthly income (USD)	4.3 (1.8)	4.1 (1.8)	*p* = .003
Subjective health state	86.3 (12.6)	86.7 (13.5)	*p* = .656
BMI	27.3 (4.9)	27.2 (4.8)	*p* = .615
Underweight	8 (2.3%)	5 (2.5%)	*p *= .873
Normal	109 (30.7%)	58 (29.0%)	
Overweight	142 (40.0%)	91 (45.5%)	
Obese	96 (27.0%)	46 (23.0%)	
Chronic illnesses (sum)	1.8 (1.2)	2.2 (1.5)	*p* < .001
Asthma (% yes)	40 (11.2%)	25 (12.5)	*p* = .727
Diabetes (% yes)	56 (15.7%)	32 (16.0%)	*p* = .999
Hypertension (% yes)	184 (51.5%)	111 (55.5%)	*p* = .180
Cardiovascular disease (% yes)	39 (10.9%)	21 (10.6%)	*p* = .999
Stroke (% yes)	13 (3.6%)	10 (5.1%)	*p* = .727
Chronic lung disease (% yes)	24 (6.7%)	24 (12.1%)	*p* = .052
Arthritis (% yes)	54 (15.1%)	27 (13.6%)	*p* = .999
Cancer (% yes)	36 (10.1%)	22 (11.0%)	*p* = .250
Liver disease (% yes)	10 (2.8%)	11 (5.6%)	*p* = .227
Osteoporosis (% yes)	142 (39.8%)	96 (48.5%)	*p* = .248
Systolic blood pressure	137.3 (20.6)	142.1 (19.0)	*p* < .001
Cardiovascular risk score	1.2 (1.0)	1.2 (0.9)	*p* = .773
Systolic blood pressure (% at risk)	164 (45.9%)	104 (52.0%)	*p* = .004
Physical activity (% frail)	117 (32.8%)	42 (21.0%)	*p* = .005
Past/current tobacco use (% yes)	85 (23.8%)	51 (25.5%)	*p* = .388
BMI classification (% at risk)	96 (27.0%)	48 (24.0%)	*p* = .999
Medications (sum)	2.5 (2.0)	2.8 (2.2)	*p* = .021
Polypharmacy status (% ≥5 medications)	61 (17.1%)	39 (19.5%)	*p* = .216
Falls			*p* = .930
No falls	259 (72.6%)	141 (70.5%)	
One fall	60 (16.8%)	38 (19.0%)	* *
≥2 falls	38 (10.6%)	21 (10.5%)	* *
Overall frailty^a^			*p* = .821
Not frail	113 (31.7%)	77 (38.5%)	* *
Pre-frail	212 (59.3%)	107 (53.5%)	* *
Frail	32 (9.0%)	16 (8.0%)	
IADL	7.9 (0.6)	7.9 (0.5)	*p* = .893
BADL	5.8 (0.4)	5.9 (0.3)	*p* = .045
Difficulty sleeping (% yes)	139 (38.9%)	67 (33.8%)	*p* = .525
Depression symptoms (GDS-15)	1.8 (2.1)	1.6 (2.0)	*p* = .146
Subjective cognitive impairment (% yes)	155 (43.4%)	106 (53.0%)	*p* = .006

M, mean; SD, standard deviation; USD, U.S. dollars; monthly income was measured using a Likert-scale (0 = ≤ 250$, 1 = 251$–500$, 2 = 501$–850$, 3 = 851$–1,200$, 4 = 1,201$–1,600$, 5 = 1,601$–2,000$,  6 = > 2,000$). BMI, body mass index; GDS-15, geriatric depression scale-15 items; IADL, instrumental activities of daily living; BADL, basic activities of daily living.

^a^ Frailty was assessed using the five components proposed by Fried.

**Table 2:** Composite scores of cognitive domains.

	Visit 1 *n, M* (SD)	Visit 2 *n, M* (SD)	*P* value
Global cognition	27.3 (1.9)	27.9 (1.6)	*p* < .001
Verbal learning^a^	0.3 (1.5)	0.0 (1.7)	*p* = .002
Long-term memory^a^	0.3 (1.6)	0.0 (1.8)	*p* = .002
Attention^a^	0.3 (1.5)	0.0 (1.7)	*p* < .001
Executive function^a^	0.6 (1.9)	0.4 (1.9)	*p* = .027
Visuospatial abilities^a^	0.0 (1.7)	−0.002 (1.7)	*p* = .794
Language^a^	0.1 (2.3)	0.0 (2.4)	*p* = .208
Processing speed (sec)	45.3 (17.5)	46.5 (19.2)	*p* = .257

M, mean; SD, standard deviation. Global cognition was measured using the raw score of the mini-mental state examination (MMSE). Processing speed was measured using the raw score of the trail making test A (seconds).

^a^ Scores for cognitive domains represent standard z scores.

Independent samples *t*-tests comparing the 157 participants who did not complete the second interview with the 200 participants who completed both interviews revealed that those who did not return were, on average, older (*M* = 70.0 vs. 68.6 years old, *p *= .041) and had lower learning (*M* = −.34 vs. .27, *p *< .001), long-term memory (*M* = −.30 vs. .25, *p *= .004), attention (*M* = −.39 vs. .32, *p *< .001), and processing speed (*M* = 55.87 s vs. 45.26 s, *p *< .001) scores than those who completed the follow-up visit.

### 3.2 Multiple regression analyses

For each statistical model, cognitive domains were regressed on to age, educational attainment, income, subjective health state, diabetes diagnosis, CVR score, symptoms of geriatric depression, and subjective cognitive decline (see Table 3).

**Table 3:** Multiple linear regression analysis by cognitive domain at time of first visit (*n *= 357).

Model	Dependent variable	*b*	Standard error	*β*	*t*
1. Global cognition (*n* = 354)					
Age		.02	.02	.05	.92
Years of education		−.03	.03	−.07	−1.14
Income*		.15	.06	.14	2.40
Subjective health state		.003	.01	.02	.36
Diabetes		−.15	.28	−.03	−.54
Cardiovascular risk		.18	.10	.10	1.79
Depression symptoms		.09	.05	.11	1.76
Subjective cognitive decline*		−.50	.21	−.13	−2.37
2. Verbal Learning (*n* = 353)					
Age***		−.06	.01	−.21	−4.20
Years of education**		.07	.02	.18	3.42
Income***		.18	.05	.19	3.60
Subjective health state		.003	.01	.03	.51
Diabetes		.16	.23	.03	.70
Cardiovascular risk**		.22	.08	.13	2.62
Depression symptoms		−.03	.04	−.04	−.69
Subjective cognitive decline*		−.38	.17	−.11	−2.17
3. Long-term Memory (*n* = 352)					
Age***		−.06	.01	−.23	−4.54
Years of education**		.07	.02	.18	3.41
Income**		.17	.05	.18	3.26
Subjective health state		.004	.01	.04	.63
Diabetes		.22	.24	.05	.94
Cardiovascular risk		.10	.09	.06	1.17
Depression symptoms		.002	.04	.003	.06
Subjective cognitive decline*		−.40	.18	−.11	−2.20
4. Attention (*n* = 353)					
Age***		−.05	.01	−.19	−3.95
Years of education***		.08	.02	.20	3.83
Income***		.19	.05	.21	3.97
Subjective health state		.01	.01	.11	2.00
Diabetes		−.34	.22	−.08	−.158
ardiovascular risk**		.22	.08	.13	2.75
Depression symptoms		.02	.04	.03	.50
Subjective cognitive decline		−.16	.17	−.05	−.94
5. Executive Function (*n* = 320)					
Age***		−.08	.02	−.23	.4.39
Years of education***		.13	.03	.24	4.31
Income*		.15	.07	.12	2.16
Subjective health state		.01	.01	.04	.63
Diabetes		−.31	.31	−.05	.98
Cardiovascular risk Depression symptoms		.17	.11	.08	1.60
		.01	.05	.01	.15
Subjective cognitive decline		−.36	.22	−.09	−1.62
6. Visuospatial (*n* = 354)					
Age*		−.03	.02	−.13	−2.36
Years of education		.01	.02	.03	.59
Income		.06	.05	.06	1.07
Subjective health state		−.003	.01	−.03	−.47
Diabetes		−.47	.25	−.10	−1.93
Cardiovascular risk		−.09	.09	.05	1.03
Depression symptoms**		−.14	.04	−.20	−3.24
Subjective cognitive decline		.14	.19	.04	.74
7. Language (*n* = 354)					
Age**		−.07	.02	−.17	−3.37
Years of education***		.11	0.3	.21	3.80
Income*		.18	.07	.14	2.47
Subjective health state		.001	.01	.004	.07
Diabetes		−38	.33	−.06	−1.16
Cardiovascular risk		.24	.12	.10	1.96
Depression symptoms		−.06	.06	−.06	−.97
Subjective cognitive decline		−.29	.25	−.06	−1.15
8. Processing speed (*n* = 353)					
Age***		.69	.18	.19	3.89
Years of education***		−1.03	.27	−.21	−3.84
Income**		−1.93	.67	−.16	−2.89
Subjective health state		.14	.09	.09	1.54
Diabetes		−3.81	3.00	−.06	−1.27
Cardiovascular risk*		−2.65	1.09	−.12	−2.42
Depression symptoms		−.83	.52	−.09	−.59
Subjective cognitive decline		1.10	2.28	.03	.49

Diabetes and subjective cognitive decline were coded as 0 = no, 1 = yes. *p < .05, **p < .01, ***p < .001.

### 3.3 Model 1: global cognition

The omnibus test was significant [*F* (8, 345) = 2.43, MSE = 1.88, *R^2^* = 0.05, *p* = 0.015] and results showed an effect of income (*β* = .14, *t *= 2.40, *p *= .017) and subjective cognitive decline (*β* = −.13, *t *= −2.37, *p *= .018) on global cognition. Higher income was associated with higher global cognition scores, and individuals who reported subjective cognitive decline scored lower on global cognition.

### 3.4 Model 2: verbal learning

The omnibus test was significant [*F* (8, 344) = 11.75, MSE = 1.53, *R^2^* = 0.22, *p* < 0.001] and results showed an effect of age (*β* = −.21, *t *= −4.20, *p *< .001), educational attainment (*β* = .18, *t *= 3.42, *p *< .001), income (*β* = .19, *t *= 3.60, *p *< .001), cardiovascular risk (*β* = .13, *t *= 2.62, *p *= .009), and subjective cognitive decline (*β* = −.11, *t *= −2.17, *p *= .031) on learning. Older age and subjective cognitive decline were associated with lower learning scores, whereas higher educational attainment, income, and cardiovascular risk were associated with higher scores on learning.

### 3.5 Model 3: long-term memory

The omnibus test was significant [*F* (8, 343) = 10.74, MSE = 1.59, *R^2^* = 0.20, *p* < 0.001] and results showed an effect of age (*β* = −.23, *t *= −4.54, *p *< .001), educational attainment (*β* = .18, *t *= 3.41, *p *< .001), income (*β* = .18, *t *= 3.26, *p *< .001), and subjective cognitive decline (*β* = −.11, *t *= −2.17, *p *= .029) on long-term memory. Higher educational attainment and income were associated with higher long-term memory scores. Older age and subjective cognitive decline were associated with lower long-term memory scores.

### 3.6 Model 4: attention

The omnibus test was significant [*F* (8, 344) = 13.09, MSE = 1.45, *R^2^* = 0.23, *p* < 0.001] and results showed an effect of age (*β* = −.19, *t *= −3.95, *p *< .001), educational attainment (*β* = .20, *t *= 3.83, *p *< .001), income (*β* = .21, *t *= 3.97, *p *< .001), and cardiovascular risk (*β* = .13, *t *= 2.75, *p *= .006) on attention. Older age was associated with lower attention scores. Higher educational attainment, income, and cardiovascular risk were associated with higher attention scores.

### 3.7 Model 5: executive function

The omnibus test was significant [*F* (8, 311) = 8.63, MSE = 1.89, *R^2^* = 0.18, *p* < 0.001] and results showed an effect of age (*β* = −.23, *t *= −4.39, *p *< .001), educational attainment (*β* = .24, *t *= 4.31, *p *< .001), and income (*β* = .12, *t *= 2.16, *p *= .031) on executive function. Older age was associated with lower executive function scores, and higher educational attainment and income were associated with higher executive function scores.

### 3.8 Model 6: visuospatial abilities

The omnibus test was significant [*F* (8, 345) = 3.95, MSE = 1.65, *R^2^* = 0.08, *p* < 0.001] and results showed an effect of age (*β* = −.13, *t *= −2.36, *p *= .019) and depression symptoms (*β* = −.20, *t *= −3.24, *p *< .001) on visuospatial abilities, such that older age and having more depression symptoms was associated with lower visuospatial scores.

### 3.9 Model 7: language

The omnibus test was significant [*F* (8, 345) = 8.82, MSE = 2.20, *R^2^* = 0.17, *p* < 0.001] and results showed an effect of age (*β* = −.17, *t *= −3.37, *p *< .001), educational attainment (*β* = .21, *t *= 3.80, *p *< .001), and income (*β* = .14, *t *= 2.47, *p *= .014) on language. Older age was associated with lower language scores, while higher educational attainment and income were associated with higher language scores.

### 3.10 Model 8: processing speed

The omnibus test was significant [*F* (8, 344) = 11.34, MSE = 20.07, *R^2^* = 0.21, *p* < 0.001] and results showed an effect of age (*β* = .19, *t *= 3.89, *p *< .001), educational attainment (*β* = −.21, *t *= −3.84, *p *< .001), income (*β* = −.16, *t *= −2.89, *p *= .004), and cardiovascular risk (*β* = −.12, *t *= −2.42, *p *= −.016) on processing speed. Years of education, income, and cardiovascular risk were associated with faster processing speed, and older age was associated with slower processing speed.

### 3.11 Repeated measures analyses of covariance

For global cognition and the seven cognitive domains, a repeated measures ANCOVA was conducted to assess changes in cognition from baseline to the follow-up while controlling for demographic and clinical factors. Age, educational attainment, income, subjective health state, diabetes diagnosis, cardiovascular risk, symptoms of geriatric depression, and subjective cognitive decline were entered as covariates.

The overall results revealed that only scores on the attention domain differed significantly between time points [*F* (1, 189) = 5.08, MSE = 3.12, *η^2^* = 0.03, *p* = .025], and a *post hoc* analysis (*p* < .001) showed that attention scores decreased significantly from baseline (*M* = .34) to follow-up (*M* = .04). No significant differences were observed between visits for the remaining domains (all *p*-values > .05).

### 3.12 Exploratory multiple regression analysis

An exploratory regression analysis was performed to examine demographic and clinical factors at baseline that predict change in attention scores over time (see Table 4). The change score was created by computing the difference between the attention scores at first visit and second visit. Change in attention was then regressed on to age, educational attainment, income, subjective health state, diabetes diagnosis, cardiovascular risk, depression symptoms, and subjective cognitive decline.

**Table 4:** Multiple linear regression analysis of the change in attention from baseline to follow-up (*n *= 198).

	*b*	Standard error	*β*	*t*
Age	.02	.01	.09	1.22
Years of education	−.01	.02	−.04	−.52
Income	−.04	.05	−.06	−.79
Subjective health state***	.03	.01	.27	3.41
Diabetes	.05	.23	.02	.22
Cardiovascular risk	−.01	.08	−.01	−.17
Depression symptoms***	.14	.04	.26	3.30
Subjective cognitive decline	−0.5	.17	−.02	−.27

Criterion variable was measured using the total score of the trail making test A and digit span (direct and inverse). Diabetes and subjective cognitive decline were coded as 0 = no, 1 = yes. *p < .05, **p < .01, ***p < .001.

Results indicated a significant omnibus test [*F* (8, 189) = 2.41, MSE = 1.11, *R^2^* = 0.09, *p* = 0.017] and showed a main effect of subjective health state (*β* = .27, *t *= 3.41, *p *< .001) and depression (*β* = .26, *t *= 3.30, *p *< .001) on the change in attention. Higher self-rated health and more geriatric depression symptoms at baseline were associated with greater changes in attention scores at follow-up.

## 4 Discussion

The main objective of this study was to examine the characteristics associated with variation in performance across cognitive domains in a sample of cognitively healthy older Panamanian women. We compared performance across seven cognitive domains at baseline and follow-up. First, a cross-sectional analysis was conducted to determine the health, clinical and social factors associated with different cognitive domains. Our cross-sectional analyses were consistent with studies on cognitive function in older women (10, 61). Older age was associated with worse performance across domains except global cognition and processing speed. Social determinants such as years of education and income were associated with most cognitive domains. Evidence shows that women experience social inequalities including lower income, lower educational attainment and limited stimulating activities that are related to diminished cognitive reserve and may explain worse cognitive outcomes (21, 23, 62). Research studies in other LAC countries reveal that education provides a larger cognitive reserve due to the cognitive stimulation and intellectual engagement (63–65). Additionally, education is often associated with better access to resources and healthcare, which can also influence cognitive health (66, 67).

Moreover, depression was associated only with the visuospatial domain at baseline. Although the relationship between depression and attention is not clear, depression has been linked to several factors that can contribute to cognitive impairment (29, 68), including changes in brain structure, inflammation, and vascular risk factors such as diabetes, hypertension, and obesity (68). Performance in nonverbal tests, such as visuospatial tasks, tends to decrease more quickly in older adults diagnosed with depression (69). Older adults with depression experience more difficulty analyzing and discriminating visual stimuli, and show deficits in perceptual organization (70). Some research suggests that visuospatial deficits are useful to determine the progression of subjects to AD (71).

Counterintuitive results were observed with cardiovascular risk showing positive associations with verbal learning, attention, and processing speed at baseline. The inclusion of women whose cardiovascular risk scores lie within healthy ranges may explain the relationships we observed between these markers of risk and cognition at baseline prior to the presence of cognitive impairment. There are different mechanisms that can explain the association between vascular alterations and cognitive decline that have been studied in cross-sectional (72, 73) and longitudinal studies (74, 75). Vascular risks, such as smoking or having a sedentary lifestyle, can provoke narrowing of the arteries, therefore reducing blood circulation and disruption of the flow of nutrients to the brain (76–78). Also, vascular alterations such as atherosclerosis are associated with lower cognitive performance (79, 80). All these vascular pathologies have been associated with deficits in memory, processing speed and executive function (73, 80). Studies in LAC countries have shown that prevalent chronic illnesses such as hypertension, diabetes, cerebrovascular disease, and obesity impact cognition and are associated to a higher risk of dementia (81, 82). Moreover, in women, hormonal imbalance can have diverse effects on cardiovascular health and cognitive function. For instance, the decrease in estrogen can contribute to cognitive decline (83, 84).

Subjective cognitive impairment (SCI) at baseline was associated with worse global cognition, verbal learning, and verbal long-term memory. Studies have shown that in preclinical stages of AD, objective cognitive deficits are not present, though individuals may note subtle changes in cognition that could be considered a risk factor of cognitive impairment (85) and may reflect early neurodegeneration (86, 87). Neuroimaging studies have reported an association between subjective cognitive impairment and brain atrophy, particularly in frontal and temporal lobes (88). This is consistent with memory deficits present in people with SCI. Currently, SCI is being studied as an intermediate state between normal cognition and mild cognitive impairment, and, therefore, can be a useful tool in early diagnosis of MCI and AD (89).

Attention was the only cognitive domain that showed significant changes over time. In addition, higher self-rated health and more depression symptoms were associated with greater changes in attention. Changes in attention in the elderly are not fully understood (90). Some studies indicate that more complex attention processes such as selective and alternating attention tend to decrease more than sustained attention (91), while other studies show an impact in all attention processes. In our sample, changes were associated with sustained attention. In cognitive impairment, attention deficits can appear before alterations in other cognitive functions, and poor performance in attention tasks is observed in preclinical phases of AD (92–94). Multiple studies have shown depressive symptoms are associated with cognitive decline (27, 28, 95). Depression impacts brain systems that can contribute to difficulties processing information (96), and has been associated with lower volume in frontal gray matter including the orbitofrontal cortex and the cingulate gyrus (97, 98). Further, greater subjective health state was associated with worse attention. One possible explanation for this result is that participants were relatively healthy at baseline. Approximately 75% of individuals rated their health above 80/100, which may have limited the range of subjective health scores, and therefore the ability to detect a meaningful association with cognition.

### 4.1 Strengths and limitations

This study has several limitations. First, participants had a higher level of education and income relative to the national and regional average, which may limit the generalizability of our findings. Second, the self-reported variables, such as depressive symptoms, physical activity, and subjective health may be subject to response bias. Nonetheless, evidence suggests that self-report provides accurate estimates of disability and disease comorbidity and predicts mortality and other clinical health measures (99). Third, the 17-month follow-up timeline may have been too short to observe noticeable changes in cognition, particularly in cognitively healthy individuals. Lastly, the sample size was limited due to participant attrition, which minimized statistical power at follow-up.

This study also has several strengths. We recruited a relatively large community-based cohort from an under-represented population, and implemented neuropsychological tests that measure diverse cognitive domains. The study also included detailed clinical interviews and several objectively measured variables, such as BMI and blood pressure (i.e., two important components of cardiovascular risk). Hence, our analyses accounted for potential sociodemographic, lifestyle, and health-related confounders known to impact cognition.

### 4.2 Conclusions

Age, income, and education level showed the most robust associations with cognition in our sample. In addition, subjective cognitive impairment and impaired cognition were observed across global cognition, verbal learning, and memory domains. Our study findings contribute to the investigation of cognitive health in older Hispanic women and may help to inform health professionals about predictors of cognitive decline in this population. Future studies with more extended longitudinal follow-up would contribute to our understanding of how social determinants of health and other biological and health-related markers shape cognitive trajectories in older women.

## Data Availability

The raw data supporting the conclusions of this article will be made available by the authors, without undue reservation.
